# Virulence, Antimicrobial Resistance and Biofilm Production of *Escherichia coli* Isolates from Healthy Broiler Chickens in Western Algeria

**DOI:** 10.3390/antibiotics10101157

**Published:** 2021-09-24

**Authors:** Qada Benameur, Teresa Gervasi, Filippo Giarratana, Maria Vitale, Davide Anzà, Erminia La Camera, Antonia Nostro, Nicola Cicero, Andreana Marino

**Affiliations:** 1 Nursing Department, Faculty of Nature and Life Sciences, University of Mostaganem, Mostaganem 27000, Algeria; qada.benameur@univ-mosta.dz; 2 Department of Biomedical and Dental Sciences and Morphofunctional Imaging, University of Messina, 98100 Messina, Italy; nicola.cicero@unime.it; 3 Department of Veterinary Sciences, University of Messina, 98100 Messina, Italy; filippo.giarratana@unime.it; 4 Istituto Zooprofilattico Sperimentale della Sicilia “Adelmo Mirri”, 90141 Palermo, Italy; maria.vitale@izssicilia.it (M.V.); davide.anza@gmail.com (D.A.); 5 Department of Chemical, Biological, Pharmaceutical and Environmental Sciences, University of Messina, 98100 Messina, Italy; erminia.lacamera@unime.it (E.L.C.); antonia.nostro@unime.it (A.N.); andreana.marino@unime.it (A.M.)

**Keywords:** virulence genes, antimicrobial resistance, extended-spectrum β-lactamases, biofilm formation

## Abstract

The aim of this study was to assess the virulence, antimicrobial resistance and biofilm production of *Escherichia coli* strains isolated from healthy broiler chickens in Western Algeria. *E. coli* strains (n = 18) were identified by matrix-assisted laser desorption–ionization time-of-flight mass spectrometry. Susceptibility to 10 antibiotics was determined by standard methods. Virulence and extended-spectrum β-lactamase (ESBL) genes were detected by PCR. The biofilm production was evaluated by microplate assay. All the isolates were negative for the major virulence/toxin genes tested (*rfbE*, *fliC*, *eaeA*, *stx1*), except one was *stx2*-positive. However, all were resistant to at least three antibiotics. Ten strains were ESBL-positive. Seven carried the β-lactamase *bla*_TEM_ gene only and two co-harbored *bla*_TEM_ and *bla*_CTX-M−1_ genes. One carried the *bla*_SHV_ gene. Among the seven strains harboring *bla*_TEM_ only, six had putative enteroaggregative genes. Two contained *irp2*, two contained both *irp2* and *astA*, one contained *astA* and another contained *aggR*, *astA* and *irp2* genes. All isolates carrying ESBL genes were non-biofilm producers, except one weak producer. The ESBL-negative isolates were moderate biofilm producers and, among them, two harbored *astA*, two *irp2*, and one *aggR*, *astA* and *irp2* genes. This study highlights the spread of antimicrobial-resistant *E. coli* strains from healthy broiler chickens in Western Algeria.

## 1. Introduction

One of the most important global challenges to public health is represented by foodborne illnesses. Healthy food-producing animals can be vectors for a wide range of commensal and pathogenic bacteria, as well as *Escherichia coli*. This microorganism can contaminate the food chain at each step, from the slaughterhouses to the food processing phases [1,2,3,4]. To date, although several *E. coli* strains are commensals, which colonize the gastrointestinal tract of humans and warm-blooded animals, and are not often disease-causing, *E. coli* represents one of the most frequent causes of several common infections in humans and animals [5]. *E. coli* clones acquiring specific virulence factors (VFs), including adhesins, toxins, invasins, etc., can cause intestinal and extra-intestinal diseases such as enteric/diarrheal disease, urinary tract infections (UTIs) and sepsis/meningitis in human hosts [6,7,8,9,10].

VFs are generally carried on phages, plasmids or pathogenicity islands (PAIs) [11] and, among microbial strains, can be vastly interchanged via horizontal transfer [12]. Given the presence of definite virulence genes, *E. coli* strains can be classified as pathogens [13,14], in particular as zoonotic intestinal pathogenic *E. coli* pathotypes (IPEC) or extraintestinal pathogenic *E. coli* pathotypes (ExPEC) based on the type of VFs present and the host’s clinical symptoms [15]. The specific “pathotypes” are grouped into enteropathogenic *E. coli* (EPEC), enterohaemorrhagic *E. coli* (EHEC), enterotoxigenic *E. coli* (ETEC), enteroaggregative *E. coli* (EAEC), enteroinvasive *E. coli* (EIEC) and diffusely adherent *E. coli* (DAEC), and can cause intestinal diseases [8,16].

Moreover, the emergence of antibiotic resistance in food pathogens represents further complications.

The wide use of antibiotics, in both animals and humans, is responsible for an increased antibiotic resistance not only in pathogenic bacteria, but also in the endogenous microflora. Resistant animal bacteria can be transmitted to humans through several routes, such as direct contact with the animal or its manure, and through contact with or the consumption of uncooked meat [17,18,19,20,21]. Given the development of combined resistance to multiple antibiotics such as the β-lactam group, including cephalosporins and carbapenems, over the last few years, the chemotherapeutic choices for enterobacteria are becoming strictly limited [22]. Resistance to cephalosporin is the result of the production of one or more types of β-lactamases, the so called extended spectrum-β-lactamases (ESBLs) [23]. ESBLs are categorized into several classes, among which the most important include Temoniera (TEM), sulfhydryl variable (SHV) and cefotaximase (CTX-M) types [24,25]. Thus, nowadays ESBL-producing Gram-negative bacteria represent a growing concern and an important challenge for chemotherapy [26]. In addition, another issue is represented by the fact that, in food factory environments, some biofilm-forming bacteria are human pathogens. Biofilms are ecosystems made up of one or more bacterial species submerged in an extracellular matrix, whose composition varies according to the colonizing species and the food manufacturing environment [27,28,29].

The zoonotic potential of *E. coli* from chicken food products is important for public health purposes [30,31]. Meat harbors different bacteria as inherent contamination and is further contaminated during handling, improper dressing, cleaning, unsanitary conditions and unhygienic practices during its commercialization [32]. Considering the factors described, the objective of this preliminary study was to examine virulence and antimicrobial resistance (AMR) gene profiles, and the ability of biofilm formation of *E. coli* strains isolated from healthy broiler chickens in Western Algeria. The Algerian poultry industry, consisting of 20,000 farmers, every year yields an average of 340,000 tons of white meat and over 4.8 billion eggs. The present Algerian poultry industry structure is the result of government development policies, which were initiated in the 1980s [33].

## 2. Results

A total of 18 presumptive *E. coli* strains were isolated from 32 fecal samples, collected from different broiler chicken farms situated in five geographic areas of Western Algeria: Mostaganem (n = 8, 25.0%), Oran (n = 6, 18.75%), Mascara (n = 6, 18.75%), Relizane (n = 6, 18.75%) and Tiaret (n = 6, 18.75%). MALDI-TOF-MS analysis confirmed the identification of all the 18 presumptive *E. coli* strains (Table 1). All the isolates were negative for the major virulence/toxin genes tested, including shiga-like toxin 1 (*stx1*), O157:H7 serotype (*rfbE*), flagellar gene (*fliC*) and attaching and effacing gene (*eaeA*), except for one *E. coli* strain positive for the shiga-like toxin 2 (*stx2)* gene (Table 1), coming from 1/7 broiler houses located in the Mostaganem area.

However, in contrast to the low percentage of virulence genes detected, all strains were shown to be resistant to at least three antibiotics most frequently used in poultry (Table 1). They were resistant to nalidixic acid (NA) (100%), neomycin (N) (100%), tetracycline (TE) (94%), ciprofloxacin (CIP) (89%), amoxicillin–clavulanic acid (AUG) (83%), trimethoprim–sulfamethoxazole (SXT) (78%), amoxicillin (AML) (72%), cefotaxime (CTX) (50%) and chloramphenicol (C) (17%). Among the strains, 10 were phenotypically confirmed to be ESBL-positive isolates. Genotypic analyses revealed that nine strains (CTX-resistant 50%) carried the *bla*_TEM_ gene and one harbored the *bla*_SHV_ gene (5.55%). Among the *bla*_TEM_-producing *E. coli* isolates, two co-harbored the *bla*_CTX-M−1_ gene (11%) (Table 2). The distribution of the percentages of ESBL isolates and the geographical area visited was 67% in Oran, 57% in Mostaganem, 67% in Tiaret, 50% in Mascara and none in Relizane.

Furthermore, an association between biofilm production and the presence of enteroaggregative genes was evaluated.

Enteroaggregative genes were detected in the ESBL-producing strains (Table 1). Among the seven strains harboring only *bla*_TEM−1_, two strains contained iron regulatory protein 2 (*irp2)* (28.5%), two both *irp2* and the heat-stable enterotoxin-1 (*astA)* (28.5%), one *astA* (14%) and another the transcription factor (*aggR)*, a*stA* and *irp2* (14%) genes. Two strains contained *bla*_TEM_/*bla*_CTXM−1_, and one had the *irp2* (50%) gene. Among all ESBL-producing strains, only one isolate, harboring *bla*_TEM_ and *irp2* genes, was a weak biofilm producer (14%) (Table 2). The remaining strains (86%) were regarded as non-biofilm producers (specific biofilm formation (SBF): 0.16–0.29).

Among the eight non-ESBL-producing strains, five (62.5%) harbored putative enteroaggregative genes: *astA* (n = 2, 25%), *irp2* (n = 2, 25%) and *astA*–*irp2*–*aggR* (n = 1, 12.5%). Moreover, one isolate (12.5%) expressed the *stx2* gene (Table 1). The non-ESBL-producing isolates were more likely to produce biofilm than ESBL-producing strains (*p* ≥ 0.001; r = 0.85). Among the non-ESBL-producing isolates, three strains were classified as moderate biofilm producers (Table 2). One harbored *astA* (12.5%), another *stx2* (12.5%) and yet another contained no virulence gene (12.5%), with SBF: 0.81, SBF: 0.76 and SBF: 0.84, respectively. The remaining strains (62.5%) were regarded as weak biofilm producers (SBF: 0.39–0.65). *E. coli* ATCC 25922 was a moderate biofilm producer (SBF: 0.81).

## 3. Discussion

The majority of *E. coli* strains are commensals inhabiting the intestinal tract of humans and warm-blooded animals and rarely cause diseases, unless they acquire VFs carried by mobile genetic elements such as bacteriophages, pathogenicity islands and plasmids [34]. Additionally, *E. coli* can form a reservoir of AMR genes that may be transferred among different bacterial species, including pathogenic bacteria for both humans and animals.

In this study, the *E. coli* strains, isolated from fecal samples of apparently healthy chickens, showed a low percentage of virulence genes, which are characteristic of shiga toxin-producing *E. coli* (STEC O157:H7) (*rfbE*, *fliC*, *eaeA* and *stx1*). Indeed, except for one *E. coli* strain, which was positive for the *stx2* gene detected at one Mostaganem farm, all the isolates were negative for the major genes encoding VFs. This finding is in accordance with previous Algerian studies describing a low prevalence of *stx* genes in *E. coli* isolates from poultry origin, i.e., a recent Algerian study showed the presence of *stx2* in only one *E. coli* isolate from broiler chickens, which had just died [35]. Another study conducted in the north of Algeria revealed the total absence of the *stx2* gene and the presence of the *stx1* gene in only two *E. coli* strains isolated from diarrheic hens and chickens [36]. Our results are also in agreement with several previous studies conducted in other countries, which concluded that the prevalence of STEC O157:H7 in broiler chickens is relatively low compared with other animal species [37,38,39,40].

However, in contrast to the low percentage of STEC virulence genes detected, all isolated strains were shown to be resistant to at least three antibiotics most frequently administered to poultry. Antimicrobial agents are being used in many countries in veterinary practice for the treatment of disease, disease prevention and growth promotion [41]. However, the indiscriminate use of antimicrobials can result in bacterial selection pressure of the intestinal microbiota of animals [19,42,43].

The high levels of resistance of the isolated strains against more than three antibiotics were not surprising given the uncontrolled use of these antibiotics in poultry in Algeria and their use without prior antimicrobial susceptibility tests. It must also be noted that the lack of legislative restrictions on antibiotic use in the poultry industry could also lead to a build-up of antibiotic resistance, i.e., they are still used in poultry feeds at sub-therapeutic dosages for growth promotion purposes (to reduce bird mortality and improve production performance). In contrast, this practice is banned in many countries, including those of the European Union, to avoid AMR diffusion in pathogenic bacteria in food-producing animals [44]. The high level of resistance recorded in this study for CTX (50%) is troubling as third-generation cephalosporins (ceftiofur) are not used in Algerian poultry production. These results are in agreement with those reported in other studies [45,46], which highlighted the emergence and persistence of ESBL-producing *E. coli* in the poultry production pyramid in many countries despite the absence of third-generation cephalosporin usage. This might be linked to the abuse and misuse of other antimicrobials (i.e., aminoglycosides, β-lactams, quinolones, macrolides, nitrofurans, phenicols, polypeptides, sulphonamides and tetracyclines) in broiler breeding or to the selection of ESBL-producing *E. coli* in broiler breeders and their vertical transmission in the poultry production pyramid [47,48,49,50]. The high levels of ESBL-producing *E. coli* isolates in Mostaganem, Oran, Mascara, Relizane and Tiaret could be explained by their horizontal transmission in broiler farms and hatcheries, as previously suggested [51], during broiler chicken transfer and likewise through national trade to several regions of the country. In addition, encoding cephalosporin resistance genes are generally placed on self-transmissible plasmids [52], which can be promiscuous and are capable of circulating among a wide variety of hosts. Despite the fact that third-generation cephalosporins are not used in Algerian poultry production, several studies highlighted their colonization in broiler chickens in the last few years [53,54,55]. The genetic background for cephalosporin resistance was diverse in those studies. Benameur et al. [54] reported the presence of the *bla*_CTX-M−1_ gene and Meguenni et al. [55] showed the presence of *bla*_CTX-M−1_ and *bla*_CTX-M−15_. Furthermore, Belmahdi et al. [53] detected the presence of *bla*_CTX-M−1_, *bla*_SHV−12_ and *bla*_TEM−1._

However, in our study, the most prevalent group was *bla*_TEM_ followed by *bla*_TEM_ and *bla*_CTX-M−1_ gene combinations and *bla*_SHV_, like the findings in a study in Turkey that demonstrated *bla*_TEM_ as the most frequent gene, followed by *bla*_CTX-M_ and *bla*_SHV_ [56].

In many other studies, multiple occurrences of the genes were also common [57], given that these genes frequently exist in large plasmids [58]. SHV and TEM were the main types of ESBL until 2000, while, in recent decades, CTX-M enzymes took their place [59].

All genes encoding resistance to macrolides, quinolones, tetracyclines, aminoglycosides, trimethoprim, chloramphenicol and sulfonamides have been associated with plasmids containing the *bla*_CTX-M_ type [60]. The association of antibiotics, and the coexistence of *bla*_CTX-M_ genes with *bla*_TEM_ or other resistance determinants, could contribute to the spread of CTX-M enzymes. Nowadays, enzymes of the CTX-M-1 group are frequently identified in North Africa [61].

This issue is further worsened by the formation of biofilm, which promotes an additional bacterial tolerance or resistance to antimicrobial agents [29,62] and represents an advantage in the survival against host defense factors, antibiotics, physical and chemical stress as well as disinfectants [63,64].

In this study, the ability of biofilm formation was found to be significantly higher in negative ESBL strains of *E. coli* than in strains carrying the *bla*_TEM_ gene. However, despite the small number of strains used in this study, the results align with those of other authors who demonstrate that the expression of the *bla*_TEM_ gene can negatively impact biofilm formation in *E. coli* [65].

The production of biofilm is also regulated by putative enteroaggregative genes such as the transcription activator known as “*aggR”*, the master regulator of EAEC virulence, which controls the expression of adherence factors, and several other genes including yersiniabactin operon (*irp2*) and EAST1 toxin (*astA*) [61].

However, according to other authors, no correlation was reported between *aggR* alone or in association with *irp2* and *astA* and biofilm formation in producing isolates, indicating that there are additional factors involved in biofilm production in EAEC [9,66,67].

## 4. Materials and Methods

### 4.1. Study Area and Sampling

A total of 16 broiler farms were randomly selected to carry out this study. All the farms were located within five geographic areas of Western Algeria, namely Mostaganem, Oran, Mascara, Relizane and Tiaret, representing the major broiler poultry producing sites in Algeria. Each broiler farm comprised several houses. Two poultry houses were sampled from each farm and one sample per house was collected. The poultry houses were chosen by considering their capacities (at least 3000 birds per house). All the farms included in this study were under control by official veterinary services. Broilers were commonly kept for a short period, which is generally less than two months. All broiler farms were visited once and sampling was carried out a few days before submission of the birds to slaughter. Fresh (still soft and warm) poultry feces was sampled from the poultry houses and transported to the laboratory for isolation of *E. coli*. All sampled broiler flocks were apparently healthy on the day of sampling.

### 4.2. Escherichia coli Isolation

Between March and September 2020, a total of 32 fecal samples, collected from different broiler chicken farms situated in five geographic areas of Western Algeria (Mostaganem, Oran, Mascara, Relizane, Tiaret), were analyzed in this study. To isolate *E. coli*, one gram of fecal specimens was mixed with 9 mL of buffered peptone water and incubated for 18 h at 37 °C. A drop was then streaked on MacConkey agar (MAC, Oxoid, Hampshire, UK) plates and incubated for 18 h at 37 °C. *E. coli* ATCC 25922 and ATCC 10536 (American Type Culture Collection, Rockville, MD, USA) were used as reference strains. Single colonies were stored in glycerol at −80 °C until further testing.

### 4.3. Identification of Colonies by MALDI-TOF-MS

The presumptive *E. coli* colonies were identified by matrix-assisted laser desorption–ionization time-of-flight mass spectrometry (MALDI-TOF-MS). Briefly, strains were cultured on tryptic soy agar (TSA; Oxoid, Hampshire, UK) supplemented with 5% of sheep blood and incubated at 37 °C for 24 h.

A single bacterial colony was deposited on FlexiMass MALDI target plates, with 48-well sample spots (bioMérieux, Firenze, Italy), followed by the addition of 1 μL of matrix of alpha-cyano-4-hydroxycinnamic acid matrix in 50% acetonitrile and 2.5% trifluoroacetic acid (Vitek MS-CHCA, bioMérieux, Firenze, Italy).

*E. coli* ATCC 8739, used as a calibrator and internal ID control, grown on TSA, which was supplemented with 5% of sheep blood (according to the constructor procedure) and incubated at 37 °C for 24 h, was inoculated on the calibration spots as well as the test strains. The prepared plate, after the complete crystallization of the microbial matrix complex, was inserted in a Vitek MS Axima Assurance linear mass spectrometer (bioMérieux, Firenze, Italy) set with a laser frequency of 50 Hz, an acceleration voltage of 20 kV, an extraction delay time of 200 ns and mass spectra from 2000 to 20,000 Da. Every single strain was analyzed three times in three separate runs at different times.

The obtained mass spectra for each microorganism were analyzed by SARAMIS software (Spectral ARchive And Microbial Identification System—Database version 4.10—Software year 2010, bioMérieux, Firenze, Italy) by comparing them with the database bacteria reference spectra. The result of this comparison, calculated by the software algorithm, is a percentage probability (confidence level) that represents the similarity (presence or absence of specific peaks) among the obtained spectra and the reference spectra.

A perfect match reported as “excellent ID” corresponded to a percentage probability of identification (confidence level) of 99.9%, a “good ID” from >60% to 99.8%, while for <60% “no identification” (no ID) was given.

### 4.4. Genes Encoding VF Detection by Polymerase Chain Reaction

All *E. coli* isolates were tested for the genes encoding VFs characteristic of pathogenic *E. coli* O157:H7: *stx1, stx2*, *rfbE*, *fliC* and *eaeA*, using specific primers [68]. Each *polymerase chain reaction* (PCR) reaction was performed in a 50 μL amplification mixture consisting of 10 μL 5 × PCR buffer (1.5 mM MgCl_2_), 5.0 μL dNTPs (2.5 mM), 1 μL of each primer (10 μM), 0.25 μL of GoTaq DNA polymerase (5 unit/μL) and 10 μL of template. *E. coli* ATCC 43894 was used as a reference strain (*E. coli* O157:H7). The sequence of the used primers and the conditions of PCR were performed according to Tabashsum et al. [68]. Amplification products were separated by electrophoresis on 1.5% agarose gel, on 1 × Tris-Acetate-EDTA (242 g/L trizma base; 57.1 mL/L glacial acetic acid; 100 mL/L EDTA 0.5 M pH 8.0) at 100 V for 1 h and then visualized by GelRed staining, illuminated by UV transilluminator and visualized by a gel reader (Bio Rad Gel DOC XR+, Hercules, CA, USA). A 100 bp DNA ladder was used as a marker for PCR assay. The expected sizes of products for *eaeA*, *rfb* O157 and *fliC* H7 gene amplification were 150, 259 and 625 bp, and for *stx1* and *stx2* genes were 348 and 584 bp, respectively [68].

### 4.5. Antimicrobial Susceptibility Testing

Antimicrobial susceptibility of the isolates was tested using the Kirby Bauer method according to the Clinical and Laboratory Standards Institute (CLSI) guidelines [69]. The following antibiotics were tested: NA, 30 µg; CIP, 5 µg; AML, 25 µg; AUG, 20/10 µg; CTX, 30 µg; TE, 30 µg; SXT, 1,25/23,75 µg; N, 30 µg; C, 30 μg; CT, 50 μg (Oxoid, Hampshire, UK). Briefly, the isolates were grown on TSA for 24 h at 37 °C. Subsequently, each bacterial suspension was adjusted to McFarland 0.5 in normal saline and uniformly spread onto Mueller–Hinton agar (MHA; Oxoid, Hampshire, UK). Paper disks impregnated with antibiotics were placed on the surface of agar plates and incubated for 24 h at 37 °C aerobically. Then, the diameters of the inhibition zones were measured by using a Vernier caliper and the values were interpreted according to the CLSI guidelines [69]. *E. coli* ATCC 25922 and ATCC 10536 (American Type Culture Collection, Rockville, MD, USA) were used as quality control strains.

### 4.6. Phenotypic Confirmation of ESBL Production

Phenotypic confirmation of ESBL production was performed by double-disk synergy test according to the CLSI guidelines [69], by positioning an AUG disk at a distance of 30 mm to third-generation cephalosporin disk (CTX) on MHA. The test was considered as positive when a synergy (champagne cork aspect) between AUG and CTX disks was observed in combination with resistance or reduced susceptibility to third-generation cephalosporin. Isolates showing decreased susceptibility to third-generation cephalosporin without clear synergy were subjected to a Combination Disk Test, by applying disks containing third-generation cephalosporin alone and in combination with clavulanic acid, following CLSI guidelines [69].

### 4.7. ESBL Gene Identification by PCR

DNA of the isolated *E. coli* strains was prepared by boiling methods. Briefly, for each strain, 2 or 3 colonies were dissociated in 1 mL of distilled sterile water and centrifuged for 5 min at 13,000 rpm. The supernatant was eliminated, and the pellet was suspended in 200 μL of distilled sterile water and heated at 100 °C for 10 min, cooled on ice for 5 min, and the DNA was removed from the supernatant after 5 min of centrifugation (13,000 rpm) to pellet the cellular debris and stored at −20 °C until use. Genetic characterization of ESBLs was performed on phenotypically confirmed *E. coli* isolates by PCR. The sequence of primers and the conditions of PCR for the detection of *bla*_ESBL_ genes were performed as described previously for *bla*_CTX-M_ genotype groups 1, 2, 8 and 9, *bla*_SHV_ [70] and *bla*_TEM_ [71]. Amplification products were separated by gel electrophoresis using a 2% agarose gel.

### 4.8. Putative Enteroaggregative Gene Detection by PCR

The isolates were also investigated for the detection of various enteroaggregative putative genes: *aggr*, *astA* and *irp2*. The sequence of the used primers and the conditions of PCR were performed according to Mohamed et al. [9].

### 4.9. Biofilm Formation Assay

All *E. coli* isolates were evaluated for their ability to form biofilm by staining assay, as described by Cramton et al. [72] with some minor modifications. Briefly, overnight cultures in tryptic soy broth (TSB) were adjusted in culture medium to 5 × 10^5^ CFU/mL and then 200 μL was dispensed into all the wells of the microtiter plate. The biofilm biomass formed in each well, after incubation for 24 h at 37 °C, was washed twice with phosphate-buffered saline (PBS), dried at room temperature, stained with aqueous 0.1% safranin solution (200 μL) for 1 min and then washed with water. The stained biofilms were dissolved in 30% (*v*/*v*) acetic acid and measured at OD 492 nm using a microplate reader. The following formula was applied to classify the biofilm formation: SBF = (AB − CW)/G, where AB is the stained attached bacteria (OD 492 nm), CW is the stained control wells containing bacteria-free medium only (OD 492 nm) and G is the cell growth in suspended culture (OD 540 nm) [73]. *E. coli* ATCC 25922 served as a positive control. TSB without bacteria was included as medium control.

The degree of biofilm formation of the isolates was classified into 4 categories: negative (SBF < 0.35), weak (SBF ≥ 0.35–0.69), moderate (SBF ≥ 0.70–1.09) and strong (SBF ≥ 1.10) [74].

### 4.10. Statistical Analysis

All experiments were performed in triplicate. Statistical data analysis was carried out using MATLAB_R2020a (MatWorks, Inc. Natick, MA, USA). A two-tailed Student’s *t*-test was applied to evaluate the mean ± standard deviation and the significant differences in the grade of biofilm formation among different strains. For each comparison between virulence or resistance genes and biofilm formation, a correlation coefficient (r) was determined via Pearson’s analysis. *p*-values of ≤0.05 were considered significant in all experiments.

## 5. Conclusions

In conclusion, our results reported a low frequency of virulence-associated genes of STEC O157:H7 in *E. coli* strains isolated from different poultry farms in Western Algeria. However, all isolates were shown to be resistant to at least three antibiotics most frequently used in poultry, and among these more than half were ESBL-positive *E. coli* despite no use of third-generation cephalosporins in Algerian poultry production. The ability of biofilm formation, which is considered a further virulent factor in pathogenic bacteria, was instead found to be higher among non-ESBL-producing strains of *E. coli*. Given that *E. coli* in chickens represents one of the major opportunistic pathogens and that it can be easily transferred from animals to humans, ESBL-producing *E. coli* represents an important risk factor for the poultry industry and human health. This study emphasizes the importance of monitoring the spread of the *E. coli* isolates that harbor virulence and antibiotic resistance genes in poultry farms, including the ones with healthy chickens, in order to prevent and control the spread of resistant bacteria and their virulence genes.

In Algeria, antimicrobials are not only used for therapeutic reasons but also for growth promotion and disease prevention. Consequently, the Algerian authorities should enforce AMR rules in order to guarantee a wise use of antimicrobials that will limit the risk of transmission along the food chain.

## Figures and Tables

**Table 1:** Characteristics of *E. coli* isolates.

*Strains*	Algerian Area	Virulence Gene ^1^	MALDI-TOF Mean Value	*bla* Gene ^2^	AMR ^3^
S13/15	Oran	*astA*	90.0%	None	NA, CIP, AML, SXT, TE, N
S14/15	Oran	*irp2*	87.4%	CTX-M-1, TEM	NA, CIP, AUG, SXT, TE, N, CTX
S2/15	Oran	*irp2*	85.1%	TEM	NA, CIP, AML, AUG, SXT, TE, N, CTX
S4/15	Mostaganem	*stx2*	87.5%	None	NA, AML, AUG, SXT, TE, N
S19/15	Mostaganem	None	93.0%	CTX-M-1, TEM	NA, CIP, AML, AUG, SXT, TE, C, N, CTX
S12/15	Mostaganem	*aggR, astA, irp2*	85.8%	TEM	NA, CIP, AML, AUG, SXT, TE, N, CTX
S25a/16	Mostaganem	*astA*	88.7%	None	NA, CIP, AUG, TE, N
S1/16	Mostaganem	*astA, irp2*	97.7%	TEM	NA, CIP, AML, AUG, SXT, TE, N, CTX
S22/15	Mostaganem	*astA, irp2*	89.4%	TEM	NA, CIP, AML, AUG, SXT, TE, N, CTX
S16/15	Mostaganem	*irp2*	92.6%	None	NA, CIP, AML, AUG, SXT, TE, N
S34/16	Relizane	*irp2*	93.3%	None	NA, CIP, N
S31/16	Relizane	None	91.2%	None	NA, CIP, TE, N
S33/16	Relizane	*aggR, astA, irp2*	90.3%	None	NA, CIP, AML, AUG, SXT, TE, C, N
S47/16	Tiaret	*irp2*	95.2%	TEM	NA, CIP, AML, AUG, SXT, TE, N, CTX
S6/15	Tiaret	None	96.5%	None	NA, CIP, AML, AUG, TE, N
S48a/16	Tiaret	*astA*	93.4%	TEM	NA, CIP, AML, AUG, SXT, TE, C, N, CTX
S19a/16	Mascara	None	92.7%	TEM	NA, CIP, AML, AUG, SXT, TE, N, CTX
S61a/16	Mascara	None	95.0%	SHV	NA, AUG, SXT, TE, N
*E. coli* ATCC 259222			99.9%		AML, AUG

^1^ *astA*, heat-stable enterotoxin-1; *irp2*, iron regulatory protein 2; *stx2*, shiga-like toxin 2; *aggR*, transcription factor; ^2^ TEM, temoniera; CTX-M-1, cefotaximases; SHV, sulfhydryl variable; ^3^ AMR, antimicrobial resistance; NA, nalidixic acid; N, neomycin; TE, tetracycline; CIP, ciprofloxacin; AUG, amoxicillin–clavulanic acid; STX, trimethoprim–sulfamethoxazole; AML, amoxicillin; CTX, cefotaxime; C, chloramphenicol.

**Table 2:** Enteroaggregative and ESBL genes and biofilm production of *E. coli* isolates.

Strains	*aggR*	*irp2*	*astA*	TEM	CTX-M-1	SHV	SBF	Biofilm Grade
S13/15			**+**				0.81	M
S14/15		**+**		**+**	**+**		0.16	N
S2/15		**+**		**+**			0.26	N
S4/15 *stx2*							0.76	M
S19/15				**+**	**+**		0.20	N
S12/15	**+**	**+**	**+**	**+**			0.18	N
S25a/16			**+**				0.62	W
S1/16		**+**	**+**	**+**			0.19	N
S22/15		**+**	**+**	**+**			0.29	N
S16/15		**+**					0.45	W
S34/16		**+**					0.52	W
S31/16							0.84	M
S33/16	**+**	**+**	**+**				0.65	W
S47/16		**+**		**+**			0.39	W
S6/15							0.59	W
S48a/16			**+**	**+**			0.22	N
S19a/16				**+**			0.24	N
S61a/16						**+**	0.27	N
*E. coli* ATCC 25922							0.76	M

*aggR*, transcription factor; *irp2*, iron regulatory protein 2; *astA*, heat-stable enterotoxin-1; TEM, temoniera; CTX-M-1, cefotaximases; SHV, sulfhydryl variable; SBF, specific biofilm formation; **+** gene presence; M, moderate (SBF ≥ 0.70–1.09); N, negative (SBF < 0.35); W, weak (SBF ≥ 0.35–0.69).
